# The applicability of human mobility scaling laws on animals—A Herring Gull case study

**DOI:** 10.1371/journal.pone.0286239

**Published:** 2023-08-02

**Authors:** Marcelina Łoś, Kamil Smolak, Cezary Mitrus, Witold Rohm, Nico Van de Weghe, Katarzyna Sila-Nowicka

**Affiliations:** 1 Institute of Geodesy and Geoinformatics, Wrocław University of Environmental and Life Sciences, Wrocław, Poland; 2 Department of Vertebrate Ecology and Palaeontology, Institute of Environmental Biology, Wrocław University of Environmental and Life Sciences, Wrocław, Poland.; 3 Department of Geography,Ghent University, Ghent, Belgium; 4 School of Environment, The University of Auckland, Auckland, New Zealand; US Geological Survey, UNITED STATES

## Abstract

With the development of sensors, recording and availability of high-resolution movement data from animals and humans, two disciplines have rapidly developed: human mobility and movement ecology. Addressing methodological gaps between these two mobility fields could improve the understanding of movement processes and has been defined as the Integrated Science of Movement. We apply well-known human mobility metrics and data processing methods to Global Positioning System (GPS) tracking data of European Herring Gulls (Larus argentatus) to test the usefulness of these methods for explaining animal mobility behavior. We use stop detection, spatial aggregation, and for the first time on animal movement data, two approaches to temporal aggregation (Next Time-Bin and Next Place). We also calculate from this data a set of movement statistics (visitation frequency, distinct locations over time, and radius of gyration). Furthermore, we analyze and compare the gull and human data from the perspective of scaling laws commonly used for human mobility. The results confirm those of previous studies and indicate differences in movement parameters between the breeding season and other parts of the year. This paper also shows that methods used in human mobility analysis have the potential to improve our understanding of animal behavior.

## Introduction

Movement is an integral part of human and animal lives. Uncovering movement patterns opens up a wealth of opportunities to better understand our world. Improvements in tracking technology over the past two decades have enabled humans to collect high-resolution data on animal mobility using miniaturized tags [1]. In parallel, the prevalence of personal devices has allowed collecting of human location data with high temporal and spatial resolution [2]. These technological advances have led to the development of two parallel disciplines: movement ecology and human mobility. Despite the similarities in data types and structures, there is a visible gap in the methods used in these disciplines. To address this problem and bridge the methodological gap between these disciplines, Miller et al. [3] and Demšar et al. [4] have proposed a step forward to bring together research on animal and human movement into an Integrated Science of Movement. Establishing the new interdisciplinary science can lead to more holistic approaches and a better understanding of movement patterns and the underlying behaviors.

In movement ecology, movement patterns are studied to understand animal behavior and their responses to environmental changes [5]. One of the main tasks is to determine space use of animals by mapping their distribution of movement through space and time to identify biologically important areas used for breeding, resting, and foraging [6, 7]. Animal tracking data can be used to examine animals habitat selection and determine conservation strategies [8, 9]. This has been done for small animals such as the Organ Mountains Colorado chipmunk (Neotamias quadrivittatus australis) [10] and larger animals such as mountain lions (Puma concolor) [11].

A common method of determining animal space use is to estimate home range, defined by Burt as an area that animals use for their normal activities such as food gathering, reproduction or caring for the young [12]. Over the years, several habitat characterizaiton and visualization methods have been developed [13]. These techniques fall into two classes: geometric and probabilistic estimators [14]. Most of the early attempts used geometric approaches such as minimum convex polygons (MCP) [15]. More recently, probabilistic methods have been developed. They allow to estimate the utilization distribution (UD), which describes the intensity with which an animal uses space [13]. Currently, the most popular probabilistic technique to estimate home ranges is a kernel density estimator [16, 17].

Research in the field of movement ecology is often concerned with identifying unique movement behaviors [4]. These behaviors can be described using a set of parameters (e.g., step length, turn angle, velocity) calculated from a movement trajectory [3]. Ecologists usually define behavioral states from observed movement trajectories using path segmentation methods (e.g., encamped and exploratory states in African savanna elephant movements [18], active and resting phases in moose trajectories [19]). In these methods, the movement path is decomposed into homogeneous segments reflecting different animal behaviors [20]. Seasonal or annual changes in animal movements can correspond to global environmental changes. Climate change has been shown to affect animal migrations, for example by delaying the arrival of golden eagles to their breeding grounds [21] or extending the time between blue whale calving and foraging places as krill populations change [22]. Understanding animal behavior can help analyze and predict the spread of infectious diseases (e.g., bovine tuberculosis in cattle populations [23] or avian influenza in poultry [24]). In addition to studying migration patterns or their impact on the spread of diseases, animal movement data can also be used to predict natural disasters. Wikelski et al. [25] compared animal movement profiles with volcanic activity in northern Italy. Using dynamic body acceleration, they estimated the daily movement patterns of three animal species (cows, dogs and sheep) and detected abnormal movement activity when volcanic activity was higher. These analyses show the potential for short-term earthquake predictions based on animal movement observations. The increasing number of animals tracked by GPS provides the opportunity to improve weather monitoring systems around the world. In [26] soaring birds, data have been used to evaluate wind speed and direction. The measurements derived from bird flights are rare and provide information about atmospheric conditions that is hard to obtain. Therefore, avian tracking data may contribute to high-resolution weather observation.

In our research, we focus on birds and their regular movements. Birds are a group of animals that move frequently and often travel long distances. Therefore, migratory birds play a key role in the transport of many pathogens and the spread of viruses [27]. These animals move mainly to obtain food, reproduce, disperse, and migrate. Especially during their migration season, they often travel thousands of kilometers (even between continents) in a short period [28]. Through the use of various technical tools (such as GPS, geolocators, Argos, ICARUS), the movements of birds can be accurately tracked in space and time [1, 29, 30] enabling discovery of new migration routes for numerous species [31]. As mentioned earlier, moving animals can transport other organisms and thus serve as vectors of disease or disperses of seeds [32]. Knowledge about animals’ migratory patterns is becoming increasingly important for predicting epidemics allowing for an understanding of how viruses spread. One of the most dangerous diseases transmitted by animals is avian influenza [33]. Newman et al. suggested that spring outbreaks of highly pathogenic avian influenza (HPAI) H5N1 in Asia, may be related to the overlap of wintering grounds between wild birds and domestic poultry [34]. The authors applied common methods such as MCP and UD to determine migration corridors for two bird species: bar-headed geese and ruddy shelduck. By overlaying HPAI H5N1 outbreaks with UDs (with a time lag of a few days to account for the incubation period of the viruses), they found a clear link between the bird occurrence and outbreaks.

Similar to movement ecology, data about people move can be used for variety of purposes. For example, understanding human movement helps control the spread of epidemics [35] or monitor transportation systems [36]. Raw movement trajectories have to be processed to identify patterns and unique behaviors from movement data in human mobility studies [37]. The first step in this process is trajectory segmentation. Raw mobility data are segmented into states—stops or movements. Furthermore, the segmented trajectory is combined with additional information to create a contextually enriched trajectory, that includes information about travel modes and trip purposes assigned to the move and stop segments respectively. A common approach for identifying stops in movement trajectories is based on spatial and spatiotemporal clustering [38]. Clustering algorithms such as k-means or density-based methods allow for spatial grouping of location data [39, 40] resulting in groups of points classified as individual stay-regions, representing the locations visited by each person. In these methods, data are clustered based on spatial information only, whereas methods such as ST-DBSCAN use spatial and temporal information to perform spatiotemporal clustering [41]. After the detection of stay-regions, the movement data are usually temporarily aggregated. This results in a discrete sequence of temporally ordered symbols, where each symbol represents a stay-region visited by a person [42]. Such a sequence represents a movement. There are two commonly used approaches for temporal aggregation of movement trajectories: the Next Time-Bin (NTB) and the Next Place (NP) methods [43].

Human mobility represented as a sequence of places and trips can be analyzed to uncover the mechanisms underlying movement (e.g., the need to explore of new locations or returns to previously unvisited locations [44]). Inferring mobility patterns from the population can be done using mobility models.

In geography and transportation, spatial interaction (SI) models are used to model human flows and movement between places. The most widely used of these models is the gravity model, whose name corresponds to Newton’s law of gravity. The basic assumption of this model is that the flow between two places is directly proportional to their population and inversely proportional to the power function of the distance between them [45]. Over the years, improvements in calibration methods for spatial interaction models have led to fully developed entropy maximization and discrete choice models that are widely used in geography and transportation studies [46]. Another commonly used spatial interaction model known as intervening opportunities makes different assumptions than gravity-type spatial interaction models. In intervening opportunities models the number of people visiting a location at a given distance is directly proportional to the opportunities at that location and inversely proportional to the number of intervening opportunities. To constraint the lack of universality of the gravity model, Simini et al. [47] presented another spatial interaction model: the parameter-free radiation model. This model states that the flow between places also depends population distribution between these two locations.

From a statistical physics perspective, it has been suggested that human trajectories are best modeled as Levy Flights (LF) or Continuous-Time Random Walk (CTRW) [48], which are a type of Random Walk (RW) models. However, mobility studies have shown that human mobility exhibits a high degree of temporal and spatial regularity in contrast to the random trajectories of LF, CTRW or Brownian motion models [49]. The statistical patterns of human mobility have been found to exhibit scaling properties [44, 48, 49]. Humans unconsciously follow a set of mobility laws such as the power-law-like motion distribution [48, 49], dwell time distributions [49] or visitation frequency distributions [44, 49]. Uncovering scaling laws leads to a better understanding of spatio-temporal phenomena of movement and enables a universal description of movement. Song et al. [44] showed that empirical observations: visitation frequency and distinct locations over time follow highly reproducible scaling laws and highlight the limitations of the CTRW model. The frequency of visits to the r_th_ most popular location follows Zipf’s law, suggesting that human visitation patterns are nonuniform, unlike the LF and CTRW models. In the case of the number of distinct locations, the tendency to explore new places decreases over time. Random walk models or Brownian Motion models do not account for the scaling properties of human mobility, which states further shortcomings of these models. Incorporating scaling laws into mobility models better captures the pattern of basic mobility features (high degree of regularity and predictability).

**Fig 1:**
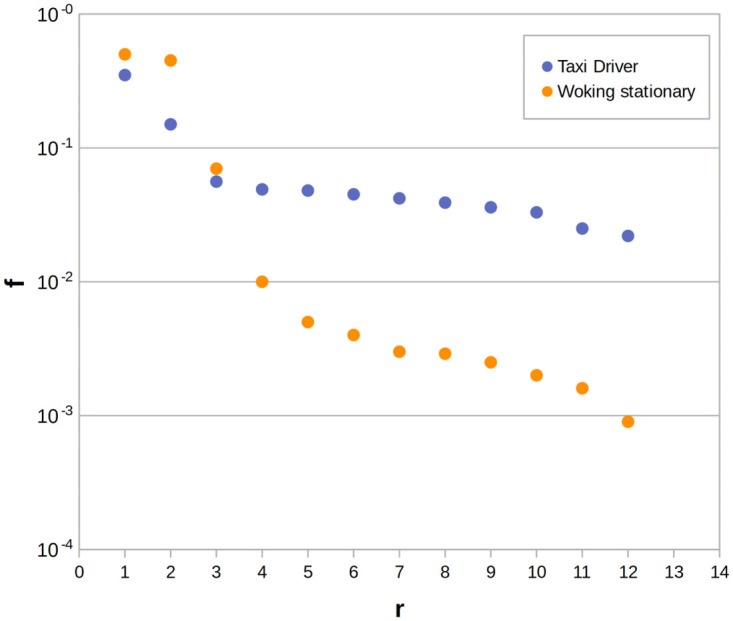
Zipf’s diagram showing the visitation frequency (*f*_*r*_) to the r-th most visited location of a user who is stationary and works as a taxi driver. A person who is stationary (orange dots) has higher (*f*_*r*_) at the first and second most visited places and for further places (*f*_*r*_) decreases significantly. For a taxi driver (blue dots) the first and second place in the ranking has lower (*f*_*r*_) and for further places the (*f*_*r*_) is higher compared to the person working stationary.

**Fig 2:**
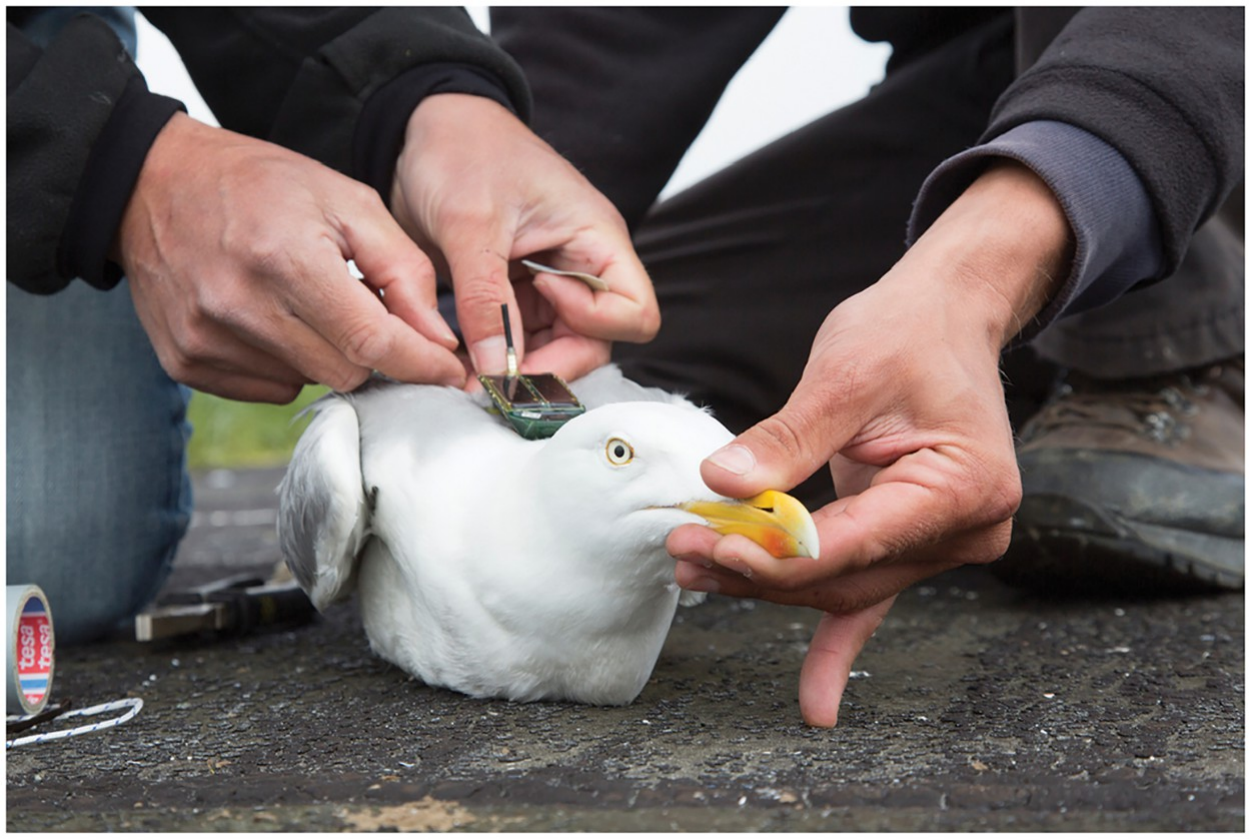
Herring Gull with a UvA-BiTS GPS tracker in Ostend. Photo by Misjel Decleer, Vlaams Instituut voor de Zee Photo Gallery (Stienen et al., 2016) [57].

In this research, we aim to search for mobility laws in animal movement using commonly applied methods to human mobility. In human data, the visitation frequency of empirical observations *f*_*r*_ and distinct locations over time *S(t)* show that trajectories follow collective scaling laws: 1) the visitation frequency at different locations decreases with the rank of preference for a single individual, similar to Zipf’s law (Fig 1); 2) there is a decreasing tendency in time to explore new locations by individuals, which means that the longer trajectory of an individual is, the more difficult it is to find a place that has never been visited. In the classical gravity model, the frequency distribution presents the probability of visitation frequency at a location. According to this law, the number of visitors to any location decreases with the distance traveled. The visitation frequency law in this paper defines a relationship between *f*_*r*_ and a ranking of locations. This scaling law varies across empirical studies, while the probability distribution of frequency generally follows a power law [50]. In [44] the exponents of *f*_*r*_ and *S(t)* are *ζ* and *μ* respectively and were calculated based on empirical data giving the value of *ζ* ≈ 1.2±0.1 and *μ* ≈ 0.6±0.1 for human mobility. In most cases, people have few frequently visited locations and visit their first and second locations in rank order, usually home and work, with similar frequency [51]. The scaling laws describe collective behavior, and thus focus on individuals, who may have show different movement patterns. A person who works stationary might have a higher visitation frequency in the most visited places (home and work). In contrast, for a person who is nor stationary (e.g. taxi driver) may have a significantly higher number of visited locations, resulting in a lower visitation frequency for these places (Fig 1). The shape of the curves describing these dependencies varies depending on the mobility patterns of the person. The *f*_*r*_ curve for a person, who spends most time in one place and therefore has lower mobility, will be steeper with *ζ* > 1.2, whereas for a person who visits numerous locations and revisits them frequently, *ζ* will be lower. *S(t)* captures the tendency to explore new places. For humans *μ* <1, it shows a decreasing tendency to of visit places not previously visited [44].

The goal of this work is to take a step toward the integrated science of movement by applying methods for aggregating spatio-temporal data and metrics to animal tracking data to investigate whether animal behavior follows scaling laws. By analysing relatively small datasets of gull and human movement data, we aim to test whether this approach can contribute to a better understanding of animals’ daily and seasonal routines and predict their behavior.

## Materials and methods

### Data and study area

The data used for this study are openly available on the Movebank data repository and come from the bird tracking dataset published by the Research Institute for Nature and Forest [52]. The birds since have been tracked using the University of Amsterdam Bird Tracking System since 2013. The dataset contains 60 individuals of European Herring Gull (Larus argentatus), tagged in or near their breeding area at the southern North Sea coast (Ostend and Zeebrugge in Belgium) (Fig 2). The European Herring Gull is a large bird (weight 800–1250 g, and wingspan of 137–146 cm) and belongs to the order Charadriiformes and family Laridae [53]. This gull species breeds mainly in northwestern Europe covering areas from southern Spain through Great Britain and Iceland, to northern Finland and northwest Russia [54]. Herring Gulls breed in colonies where each female lays 2–3 eggs that are incubated for about 30 days [54]. After another 35–40 days the young leave their nests and become independent after the next 20 days [55]. After the breeding season individuals of coastal populations gather in flocks and stay in a wide strip of European coasts, only sometimes flying deep inland [56].

**Fig 3:**
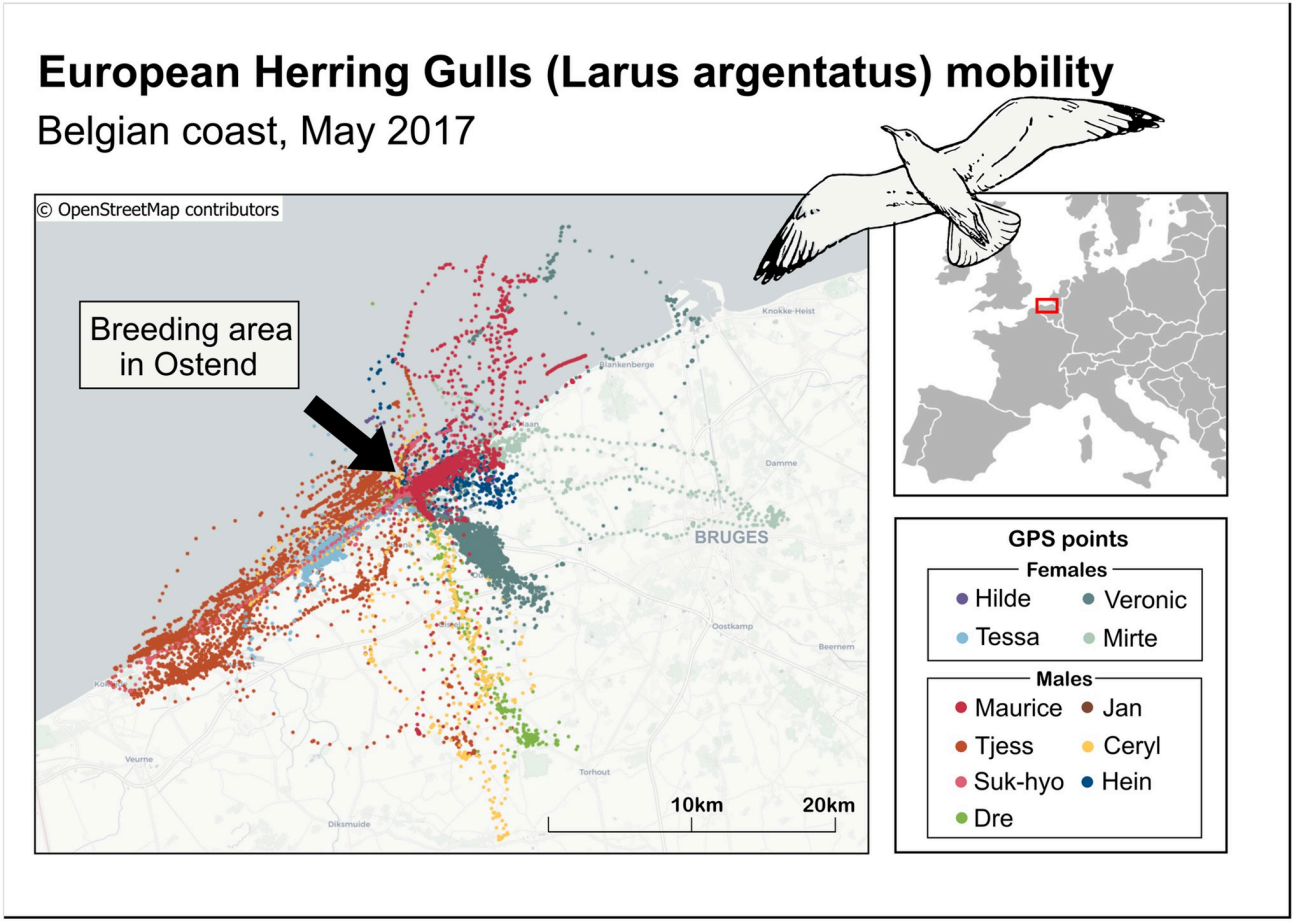
Herring Gull (Larus argentatus) mobility. Belgian coast, May 2017. Inset of European countries contains public sector information licensed under the Open Government License v3.0.

Raw data consisted of 2-dimensional location coordinates collected during the day and night at irregular intervals, 2 minutes on average. In order to have continuous coverage of data, we selected 11 Herring Gulls (4 females, 7 males) covering a period from January to November 2017 (Table 1). The mobility of selected gulls in May 2017 is shown in Fig 3.

**Table 1:** Herring Gull selected for analysis.

*id*	*name*	*sex*	*id*	*name*	*sex*
900926	Hilde	female	903622	Tjess	male
905324	Veronic	female	903149	Ceryl	male
905201	Tessa	female	903134	Suk-hyo	male
903297	Mirte	female	903132	Hein	male
905203	Maurice	male	903128	Dre	male
903644	Jan	male			

### Human data for comparison

As this study aims to verify the use of human mobility metrics and processing methods on animal data, a human mobility dataset with similar sample size was processed and used for comparison. We use data collected via smartphones in the area of London, UK. The data cover month-long movement trajectories from 11 people from a large urban area. The data contain location coordinates collected at irregular intervals (5 minutes on average). These trajectories represent a randomly selected sample. The only requirement is that the data be complete with at least 50% of the data points in hourly intervals. The data were recorded from January to March 2020. For privacy reasons, the data are not mapped.

### Data processing

**Fig 4:**
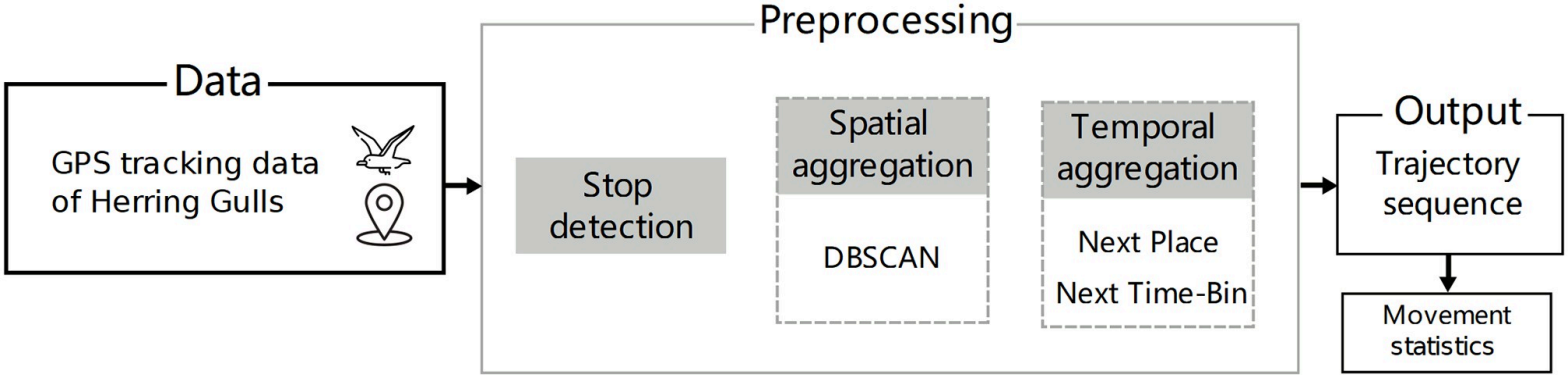
Scheme for data processing. The preprocessing of the raw data consisted of three steps: stop detection, spatial aggregation, and two approaches of temporal aggregation (Next Time-Bin and Next Place). As a result, we obtained a trajectory sequence, based on which the movement statistics were calculated.

The raw GPS data of each Herring Gull were processed into the trajectory sequence, which is a time-ordered sequence of symbols, where each symbol represents a location visited by a gull (Fig 3). These trajectory sequences were divided into months rather than season-related periods to simplify the approach and make it comparable between animals and humans. One month of raw human movement data was processed in the same way and used for comparison purposes. The scheme of this process is presented in Fig 4 and the methods used in this work are implemented by us as a part of a python-based human mobility library (HuMobi)[58].

**Fig 5:**
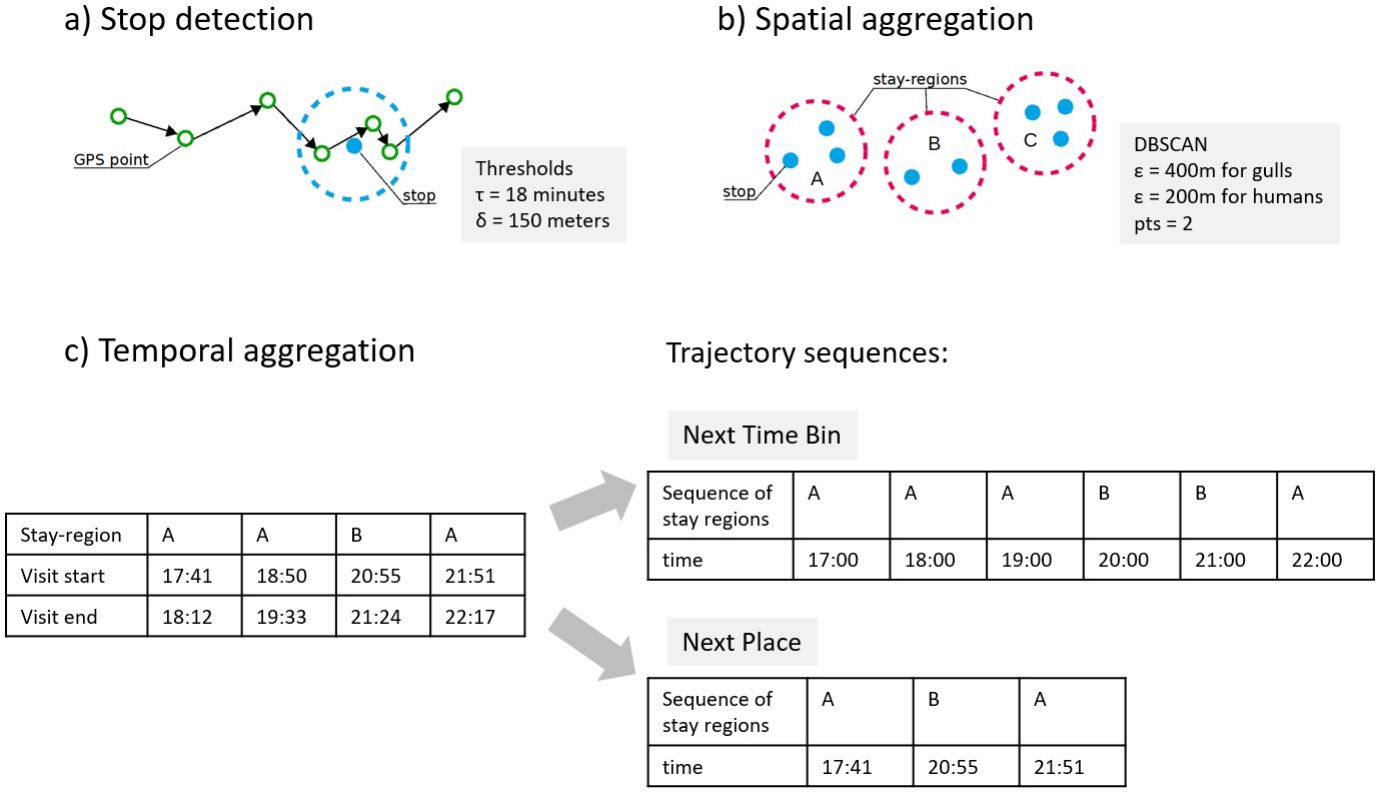
Three steps of data processing. a: Stop detection. b: Spatial aggregation. c: Temporal aggregation.

In the first step of data processing, we detected stops (locations where animal/human stops for a certain time) (Fig 5a). The stop detection algorithm starts with the first point in a trajectory (Fig 5a) and iterates through every chronologically ordered point, each time calculating the distance between the current point and the first point in the trajectory. If the computed distance is less than *δ* meters, then the current point is classified as a currently processed stop. When the distance exceeds the threshold then the time interval between the first and the previously processed point in a processed stop is calculated. If this time interval is greater than *τ* minutes then the process of detecting stop ends and a new stop-point is created from all points assigned to a currently processed stop. If the time interval is less than the threshold, all points of this stop are discarded and the process starts again from the current point in the trajectory. In our work, based on a sensitivity analysis, where we checked the number of detected stops for given *δ* and *τ*, we set *δ* to 150 meters and *τ* to 18 minutes.

Further, using the DBSCAN algorithm, the stop-points were aggregated spatially, by calculating the clusters of these points, to create stay-regions. (Fig 5b). A stop is assigned to a cluster if the distance between it and all other stops is less than *ϵ*. A stay-region is formed if the cluster contains at least two stop-points. Based on a sensitivity analysis, checking the number of detected clusters, we set *ϵ* = 400 meters for gulls and *ϵ* = 200 meters for human data.

Next, the sequences of stay-regions were temporally aggreagated using two approaches: the Next Time-Bin (NTB) and the Next Place the (NP) (Fig 5c). In NTB, a region is periodically detected at a selected time interval, which means that there can be so-called self-transitions in sequences (Fig 5c). When a gull/human has visited more than one place in a selected time interval, the location with the longer duration of stay is selected. When a gull/human visited only a few places with the same visiting time in the time interval, the location with a higher frequency of visits is selected. We one hour as the time interval because this is the most commonly used value in the literature [59]. In NP, the repeating locations were removed from the sequence of each gull/human. As a result, the NP sequence contained only transitions between stay-regions.

### Movement characteristic

The statistical characteristics of human mobility were quantitatively described using three metrics [44], that we applied to our processed datasets to test whether gull movement has similar characteristics to human mobility. Testing the usability of these metrics and turning them into curves rather than single-point-in-space measures allows for looking into unseen aspects of animal behavior.Through comparison of these curves to those obtained from human mobility analysis we can verify similarity of human and animal mobility characteristics but also suitability of these methods to animal mobility analysis. These metrics are:

- Radius of gyration—captures the average distance from the center of mass of a trajectory.
  $$\begin{array}{c} r_{g}=\frac{1}{N}\sum_{i=1}^{N}\left(r_{i}-r_{0}\right) \end{array}$$ (1)
  where r_i_ are the coordinates of the N individual points and r_0_ is the position vector from the center of mass of the set of points. In [49] radius of gyration was calculated using mobile phone call records and was suggested to be common mobility measure describing the distance typically traveled by each individual.
- Frequency of visit—corresponds to the ratio of visits to a stay-region to the number of visits in all detected stay-regions during the observation period. For human mobility, the frequency f of the r_th_ most visited stay-region follows Zipf’s law [44]:
  $$\begin{array}{c} f_{r}∼r^{-\zeta } \end{array}$$ (2)
- Distinct locations over time—S(t) shows the total number of locations visited within the time interval. In human mobility S(t) is expected to follow [44]:
  $$\begin{array}{c} S\left(t\right)∼t^{\mu } \end{array}$$ (3)

One of the goals of this research was to look at the animal data from the perspective of the collective scaling laws that are used in human mobility [44]. For the gull data, we determined *ζ* and *μ* by calculating the curve factor for every month separately. For comparison, we determined *ζ* and *μ* for the human data as well. This is the first attempt where these laws are tested for animal data and compared with human patterns. As the laws hold for a small sample of individuals for human data (see Song et al. [44], for a comparison), we are confident that choosing a small set of animals is reasonable [60].

We also tested the results for statistical differences in behavior (*r*_*g*_,*f*_*r*_ and *S*(*t*)) between female and male gulls, their behavior during breeding and non-breeding season, and for possible differences between the mobility behavior of gulls and humans. The tests used are suitable for extremely small sample sizes. Kruskal-Wallis is a non-parametric alternative to one-way ANOVA that tests whether the means of two groups are equal [61]. The Kolmogorov-Smirnov KS test is a non-parametric test that checks whether any two distributions are identical [62].

## Results

### Mobility metrics for animal movement data

**Fig 6:**
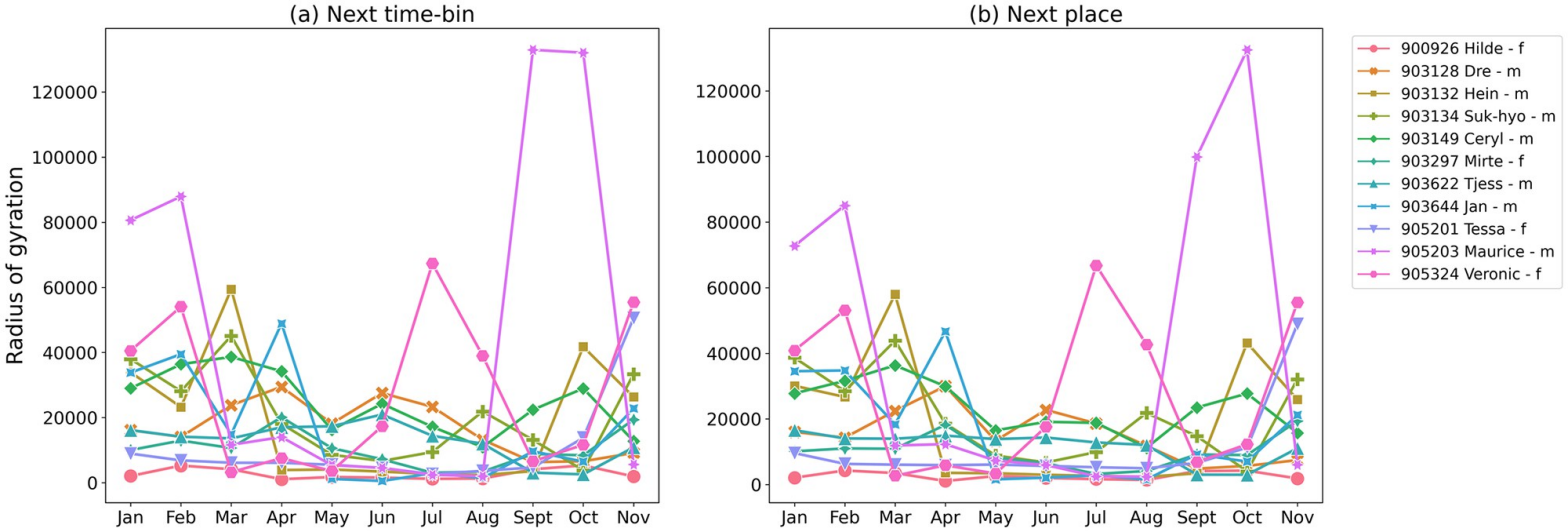
Radius of gyration *r*_*g*_ for all recorded gulls. Each animal is color-coded. The left panel (a) shows a radius of gyration for Next Time-Bin approach, while the right panel (b) shows the Next Place solution.

Fig 6 shows monthly changes in *r*_*g*_ for each gull. The gulls in this study exhibit different types of mobility, which can be shown using spatial and temporal analyses of their trajectories.

Hilde (900926) has the smallest average *r*_*g*_ of all the birds studied and stays near the port of Ostend every month. The flight paths of Tessa (905201) show a similar pattern from January to October when her movements are restricted to an area around Ostend, but at the end of October, she changes her location and flies for 70 km to Blaringhem (France). In November she moves back and forth between Ostend and Blaringhem several times. Another example of a gull with a smaller average *r*_*g*_ is Tjess (903622). In most months (January to August and November), she flies between Ostend and Nieuwpoort (17km), and for two months (September and October) her movements are restricted to the Nieuwpoort area. Maurice (905203) has the longest average flight duration and the largest *r*_*g*_, in winter (January and February) and autumn (September October) his flights are over 100km flying along the French and Belgian coasts. From March to August his flights are shorter (max. 15km) and most of his time is spent in Ostend. The longest flight distance for Dre (903128) is around 20km at the beginning of the year. From March to August he covers longer distance and occasionally flies to Blaringhem (70km). At the end of the year he increases his travel distance and flies between Zedelgem and Ichtegem (10km). The large differences the in mobility behavior of individual gulls are common as these animals easily adapt to foraging opportunities [63].

Analyzing the *r*_*g*_ chart (Fig 6) we notice the monthly mobility for gulls decreases significantly in the spring months (April-June). The lower *r*_*g*_ means gulls do not move far from their most visited location during these months, which coincides with the breeding season. These differences are statistically significant for *r*_*g*_ for the NP approach.

To evaluate the potential of scaling laws statistics for movement ecology we calculated the exponents *ζ* (2) and *μ* (3) for the study period for gull data using the NTB and NP approaches (Table 2). The results showed significant differences in mobility between breeding and non-breeding months but no significant difference in mobility behavior between the sexes of the birds (Table 3).

**Table 2:** The values of ζ and μ factor for Jan-Nov 2017.

*Month*	Jan	Feb	Mar	Apr	May	Jun	Jul	Aug	Sept	Oct	Nov
*ζ* (*NTB*)	2.20	**2.04**	2.11	2.74	**3.27**	2.96	2.39	2.21	2.21	2.30	2.15
*ζ* (*NP*)	2.05	1.99	1.93	1.89	**1.88**	1.95	**1.88**	1.91	2.03	**2.19**	2.01
*μ* (*NTB*)	0.46	0.43	0.42	0.41	**0.32**	0.40	0.43	0.44	0.47	**0.52**	0.49
*μ* (*NP*)	0.45	0.43	0.40	0.40	**0.30**	0.40	0.42	0.44	0.47	**0.51**	0.48

**Table 3:** Results of significance tests using Kolmogorov-Smirnov and Kruskal-Wallis tests.

*Groups*	*Kolmogorov* − *Smirnov test*	*Kruskal* − *Wallis test*
Breeding vs non-breeding		
NP *f*_*r*_	✓	✓*
NTB *f*_*r*_	✓	✓*
NP *r*_*g*_	—	✓*
NTB *r*_*g*_	—	—
NP *S*(*t*)	✓	✓
NTB *S*(*t*)	✓	✓
Male vs Female		
NP *f*_*r*_	—	—
NTB *f*_*r*_	—	—
NP *r*_*g*_	—	—
NTB *f*_*r*_	—	—
NP *S*(*t*)	—	—
NTB *S*(*t*)	—	—
Gulls vs Humans		
NP *f*_*r*_	—	✓
NTB *f*_*r*_	—	✓
NP *r*_*g*_	✓	✓
NTB *r*_*g*_	✓	✓
NP *S*(*t*)	✓	✓
NTB *S*(*t*)	✓	✓

✓ corresponds to the significance level <0.05 and * to <0.1

**Fig 7:**
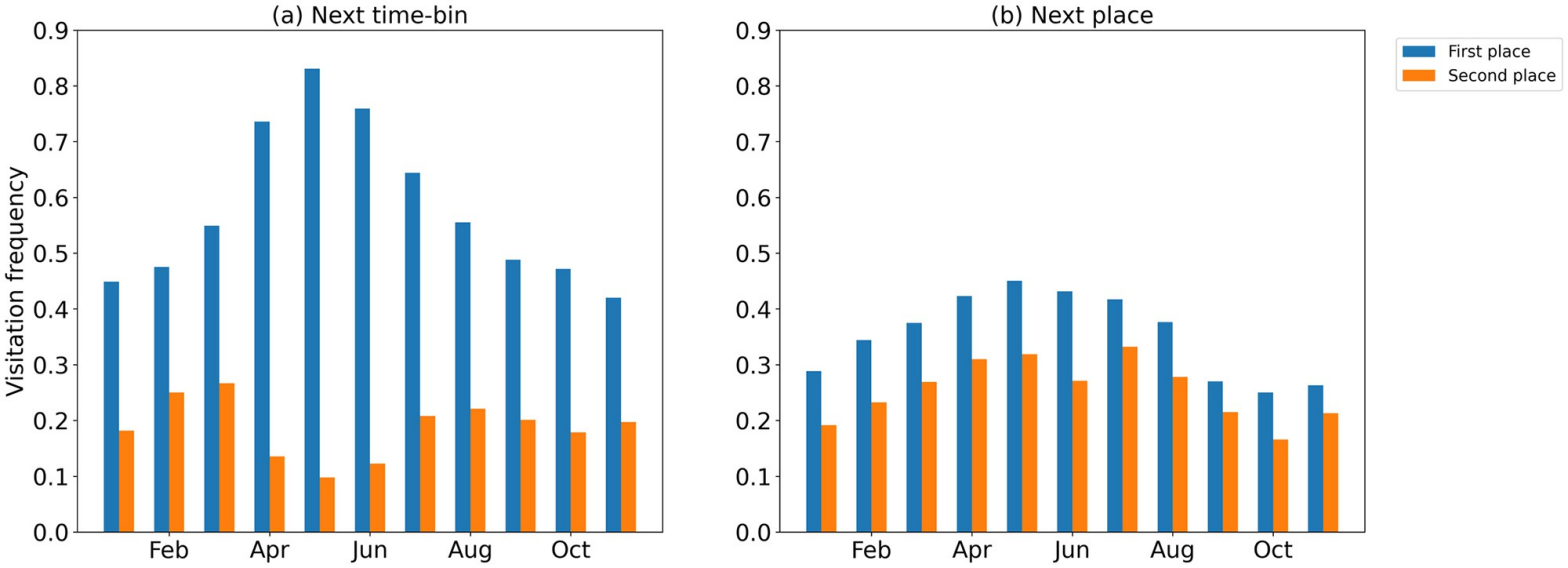
The average visitation frequency. The figures show the average visitation frequency for the first (blue) and second (orange) most visited location during the selected months for the NTB (a) and NP (b) approaches.

The radius of gyration characterizes the typical distance traveled by an individual. Fig 7 shows the monthly averages *f*_*r*_ for the first two most popular stay-regions. The results presented in these figures indicate a peak in the breeding season for the first place in the rank for NTB (Fig 7a) and NP (Fig 7b). There is a large difference in the values of *f*_*r*_ for NTB and NP which can be attributed to self-transitions in NTB sequences. The presented values of *f*_*r*_ show that individuals during the breeding season spend much more time in breeding areas but also tend to visit the second most popular region for shorter periods of time but with a similar frequency of visits. These differences between *f*_*r*_ are significant in breeding and non-breeding months (Table 3).

In the NTB, the average *f*_*r*_ for the main location from April to June is higher than 70% and in May it reaches its peak with more than 80%. In the NP approach the values of *f*_*r*_ for the first place in rank are lower than in NTB, however, they both have a tendency to increase *f*_*r*_ in the breeding season. The differences in average *f*_*r*_ for breeding and non-breeding seasons are statistically significant. For female birds during the breeding season the *f*_*r*_ is changing in the middle of May, when they start visiting more places (S1 Fig). No such change is observed in males (S2 Fig). On the other hand, the *f*_*r*_ in NTB during the breeding season differs between the sexes (S3 and S4 Figs). In males, the visitation frequency for one place is usually around 80–90% (S4 Fig), while in the females it reaches 55–70% (S3 Fig). These differences in behavior between the sexes are not significant which likely due to a small sample size (7 female and 4 male birds) (Table 3).

The differences between these two approaches (NP and NTB) are caused by self-transitions. In the NP approach trajectory sequences consist only of changing stay-regions, whereas in the NTB approach, the same stay-regions may occur several times in the same sequence resulting in self-transitions (Fig 5c). The elimination of self-transitions makes NP a better approach to reflect the characteristics of the movement, especially parameter *f*_*r*_ as the values of these metrics are artificially inflated for NTB. The second difference in temporal aggregation methods is the number of detected stay-regions for each sequences. In NTB, if visits are shorter then the time interval (onehour in our case), only one place is selected resulting in a less unique sequence compared to the NP approach.

Considering the mobility of gulls shown in Table 2, the value of *ζ* in May reaches the maximum value (3.27) for NTB, while it reaches the minimum value for NP in the same month. This effect is due to the difference between the NTB and NP sequences. Visitation frequency curve in NTB sequences is steep because gulls stay in one place for the most of the time during the breeding season, which increases the number of visits to the most frequently visited place due to self-transitions. However, as gulls travel less, in the NP sequences the most frequently visited place will be visited rarely, because gulls rarely leave that place at all. This confirms the results of previous studies [64, 65]. In terms of *S(t)*, we can see that *μ* factor decreases from March to July in NP and in NTB, which also reflects the fact that Herring Gulls tend to travel less and visit fewer unique locations during the breeding season. These results are statistically significant (Table 3).

**Fig 8:**
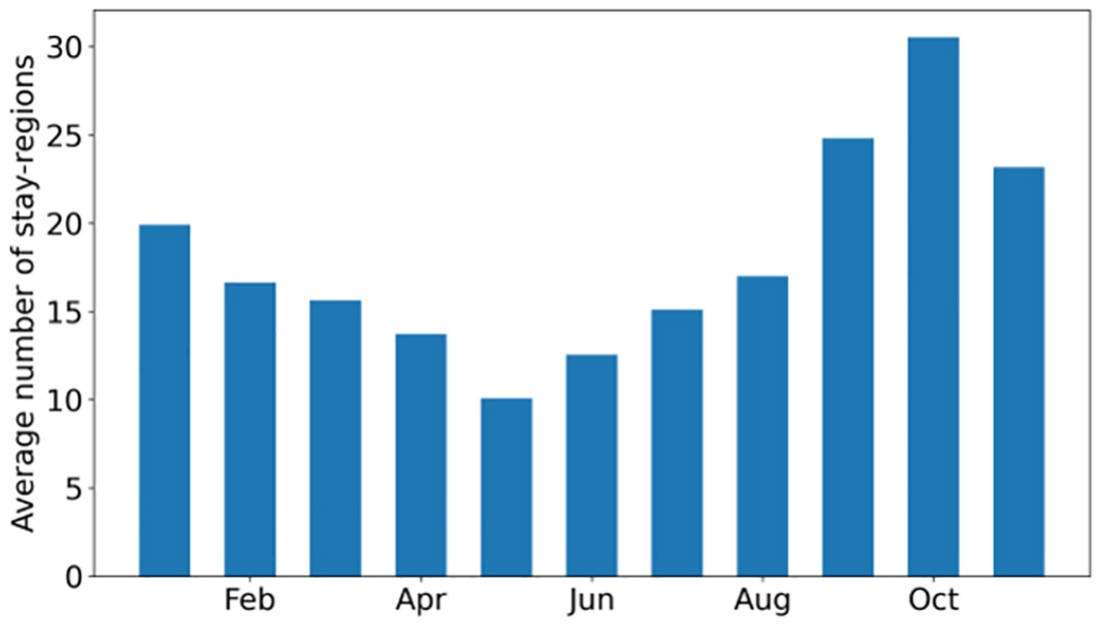
The average number of stay-regions per month. The breeding season is characterized by a decrease in the average number of visited places.

Fig 8 shows the average monthly number of stay-regions. This number is lower from February to August compared to the rest of the year (Fig 8). In May the average number of stay-regions is minimal. In September and October, the number of visited places increases, and on average gulls in our study visit 30 stay-regions per month. The changes in the number of stay-regions could reflect the phenology and life stage individual gulls. In April, during the breeding season, birds mainly stay near the breeding colony, while in September and October they move around in search of suitable foraging places abundant in food [64, 65]. In this study, there was no clear sex difference in their behavior and we could see how variable the birds studied are in terms of activity spectrum: *f*_*r*_, *r*_*g*_ etc. Some are more terrestrial and others more marine which is consistent with the results of other studies with a small sample size of birds [63].

### Comparison with human mobility data

Applying preprocessing methods and metrics commonly used in human mobility studies allows for studying animal movement from a new perspective. To test their usefulness, we processed the data and calculated a number of metrics for both human and animal data. We also tested whether there are differences in behavior between humans and animals (Table 3).

**Fig 9:**
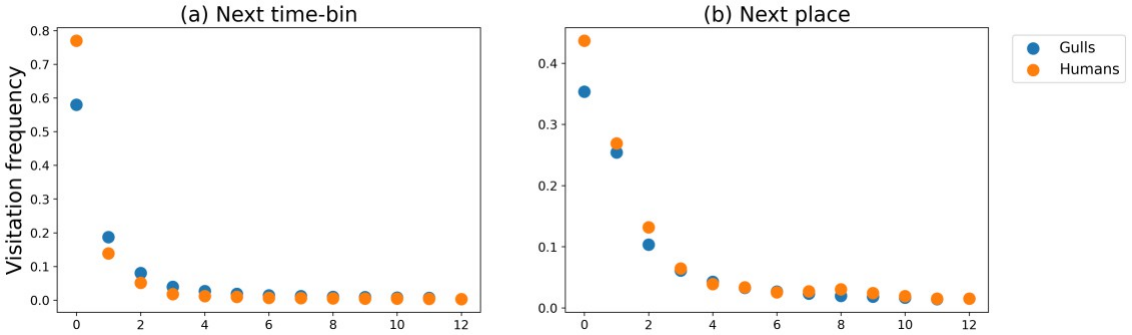
Zipf’s plot showing the visitation frequency (*f*_*r*_) of the r-th most visited location of people and Herring Gulls for NTB (a) and NP (b) approaches. The blue points correspond to gull data and the orange points to human data.

The results show that human and animal movement data scale similarly, showing comparable dependencies in the number of different locations visited or visitation frequency. The curves of *r*_*g*_, *f*_*r*_ and *S*(*t*) for animals and humans follow similar distributions. This proves that the movement behavior of Herring Gulls and humans can be studied with the same metrics. There are significant differences in the values of the calculated metrics derived from animal and human data. These result from differences in behavior between the two species. Gulls seem to have much higher total number of locations visited per month and spend potentially less time in these visited regions. For humans, *f*_*r*_ in the NTB approach is significantly higher for the first four, most frequently visited stay-regions (Fig 9). This is consistent with the fact that humans are less willing to explore unfamiliar areas and more inclined to visit the same locations for longer periods of time. The difference in mobility behavior between gulls and humans may also be related to the constraints of the urban environment and the structured lives of humans.

## Discussion

Previous studies on bird movement were primarily related to spatial analyses that focused on the places used by birds at different times and the distances traveled to migrate [1, 56]. This work, using commonly applied human data processing methods (NTB and NP approaches), focuses on studying the frequency of change and number of places visited by birds. The results show variability in bird activity over time. Studying a sample of the Herring Gull movement data allowed us to see changes in mobility patterns derived using mobility metrics over nearly the entire annual cycle (11 months). We found that mobility of birds is reduced from May to the end of June coinciding with their breeding season (Fig 8). A more detailed analysis (S1–S4 Figs) of this period indicates a decrease in movement activity during the first stages of the breeding season (April and May) which corresponds to the construction of the nest, laying, heating and protecting the eggs [56]. In the next period (June) there is a slight increase in activity by the adults, which is due to obtaining food for the developing chicks. During this period the adults forage within 20 km of their colony [64]. After the end of the breeding season, mobility increases and birds change their flight patterns and the locations they visit (Fig 9). Some birds stay near the breeding area but others leave the colony and move to other suitable feeding areas. Similar patterns are seen in the results from [55] and can be clearly identified on Fig 7. During migration and nomadic stay which lasts until the end of January, the birds are very active and often move from a region to region [64]. In early February, gulls return to their colonies and prepare for the breeding season, so their mobility decreases and they tend to stay longer in one place [65].

The methods used in this work allow the analysis of different activity parameters and can potentially reveal different mobility patterns between sexes. In our case, comparison of the frequency and number of places visited between male and female gulls shows no visible or statistical differences. Results could vary with large numbers of animals, but the lack of visible differences in this research is consistent with the results of previous studies [63, 66]. This could be due to the reproductive biology of the species, where both sexes are equally involved in incubating and caring for their chicks [67]. However, the NTB approach showed that male birds recorded a greater proportion of visits to a place, especially during the breeding season. This could be associated with territorialism which is understood as a defence of territory and nesting place by male birds [68]. This is reflected in NTB, as this approach, self-transitions are not eliminated. The visitation frequency in NTB is calculated based on one-hour intervals. If a gull spends in its most visited location few hours in a row, every hour will be treated as the next visit increasing the visitation frequency of this place. Another explanation for these potentially visible differences between the sexes may be the differences is size and preference for food competition between female and male birds. Females,being smaller and less aggressive, can fly farther and spend more time searching for food. Birds are ideal for studying dynamic changes in movement. They are characterized by a high metabolic rate, and require a constant supply of energy, associated with frequent food intake and movement [69].

The animal movement seems to scale similarly to human movement, and the dependencies and trends seen in human data are also found in the data from Herring Gulls. As the scaling laws for animal and human data follow similar patterns, there is a precedent for using human mobility metrics and more complicated methods such as spatial interaction models to study animal movement behavior. Spatial interaction models have rarely been used for animal movement studies because in most cases, we only have a small sample of individual animal movement trajectories and not collective data from the entire population [70]. With ever increasing samples of animals studied, these models could be used to explain why movements occur and to study resource use and availability in different places/ecosystems. Resource variability across landscapes will affect the movement of animals [70]. Animals will forage more frequently in closer locations with resources than in distant areas without resources. Similarly, humans plan their shopping or working strategies [71].

## Conclusion

To our knowledge, this is the first application of scaling laws for human mobility to animal movement data. To explore animal movement behavior, in this work we tested three commonly used human mobility metrics: radius of gyration, frequency of visits, and distinct locations over time, using two types of temporal aggregation: Next Time-Bin and Next Place. We also analyzed the metrics in two time scales: monthly and pentads, separately for male and female birds and compared these results to metrics derived from a sample of human mobility data.

The two key metrics in human mobility: 1) the visitation frequency and 2) distinct locations over time for investigated Herring Gulls represent similar characteristics as human mobility. The visitation frequency follows Zipf’s law, while the number of distinct locations over time shows a downward trend, just as in the case of human mobility. The shapes of the curves follow the same distributions for animal and human data but there are clear differences in the significance and the time spent in visited locations. As observed for the NTB sequences, the values of *ζ* and *μ* are close to those observed for human mobility only during the non-breeding season. During the breeding season stationary mobility behavior of gulls increases *ζ* and decreases *μ*. We found that based on the small sample size of studied Herring Gulls, mobility metrics vary throughout the year and are not sex-dependent.

As far as we know, no scaling laws have yet been calculated using the NP problem formulation, which seems to have an effect on the values of *ζ* and *μ*. The *ζ* values of visitation frequency are lower for the NP problem formulation, which is caused by the fact that all self-transitions are removed from these sequences, hence there are fewer observations at the locations that are visited for long periods of time. The *μ* values are usually higher for the NP problem formulation as this is determined by the number of unique locations present in the movement trajectories and the NTB approach tends to remove the locations that are visited for short periods of time.

This research has its limitations. The experimental dataset used in this study is relatively small and these observations would need to be verified and tested for a larger set of animal movement trajectories for multiple species. Moreover, the effects of non-breeding and breeding seasons are important. Therefore, data in future studies should be partitioned based on long-term characteristics of mobility behavior rather than by month to minimize the effects of rapid changes in mobility.We are not claiming that the scaling laws are there to replace the current and commonly used methods.They are there to add new insights into animal behavior but incorporating them to other mobility metrics widely used for animals would allow for comparison and more comprehensive analysis.

The results of this study show that it is a possible to apply methods widely used to predict and model human mobility to animal movement data. The movement metrics *f*_*r*_ and *S(t)* used in our study are explained by Exploration and Preferential Return (EPR) model. The application of EPR models to animal data can be useful in monitoring the spread of diseases such as Avian Influenza and can help control disease outbreaks. However, there are some differences in animals movement that would need to be explored in more detail to avoid potential bias in these applications.

This study used the theoretical discussions from Miller et al. [3] and Demšar et al. [4], and empirically and successfully tested the applicability of widely used human movement scaling laws to gain insights into animal behavior.

## Supporting information

S1 Fig Average visitation frequency for female gulls during the breeding season (NP). (TIF)

S2 Fig Average visitation frequency for male gulls during the breeding season (NP). (TIF)

S3 Fig Average visitation frequency for female gulls during the breeding season (NTB). (TIF)

S4 Fig Average visitation frequency for male gulls during the breeding season (NTB). (TIF)
