# Supplementary figures and images for: The applicability of human mobility scaling laws on animals—A Herring Gull case study
Source: PLoS One. 2023 Aug 2;18(8):e0286239. doi: 10.1371/journal.pone.0286239 (PMC10395819; doi:10.1371/journal.pone.0286239)

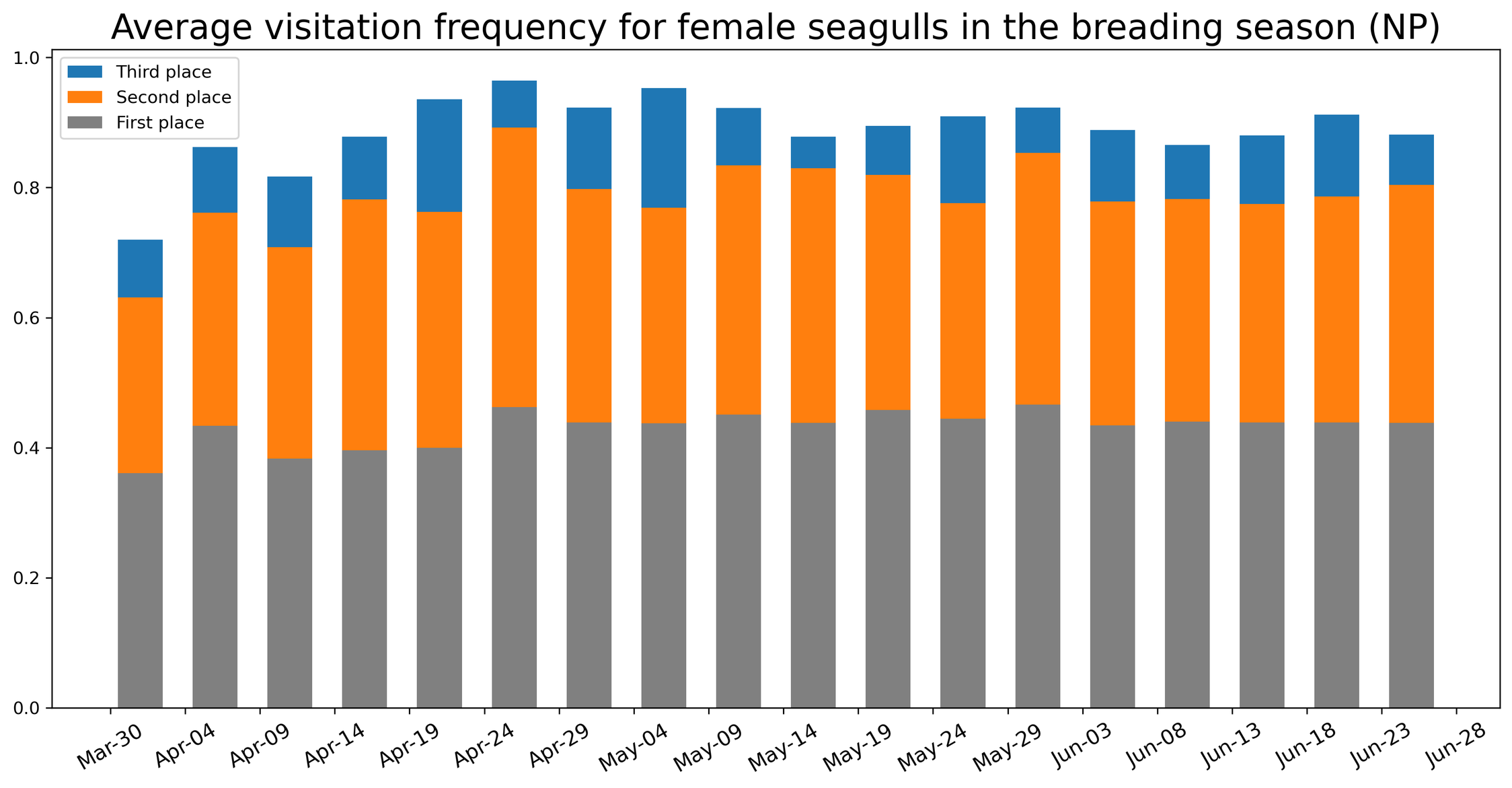

Supplement: S1 Fig — (TIF) [file pone.0286239.s001.tif]

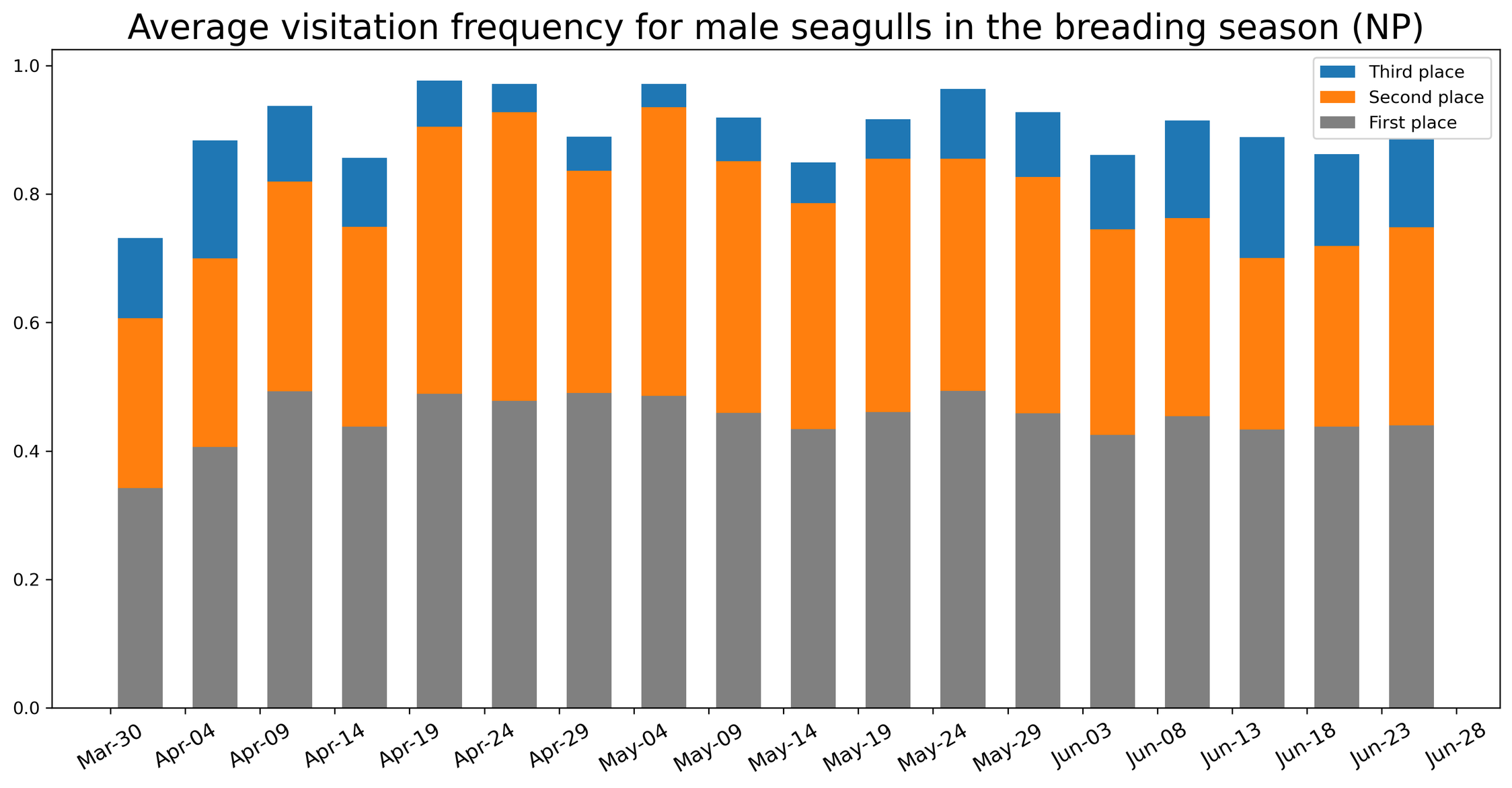

Supplement: S2 Fig — (TIF) [file pone.0286239.s002.tif]

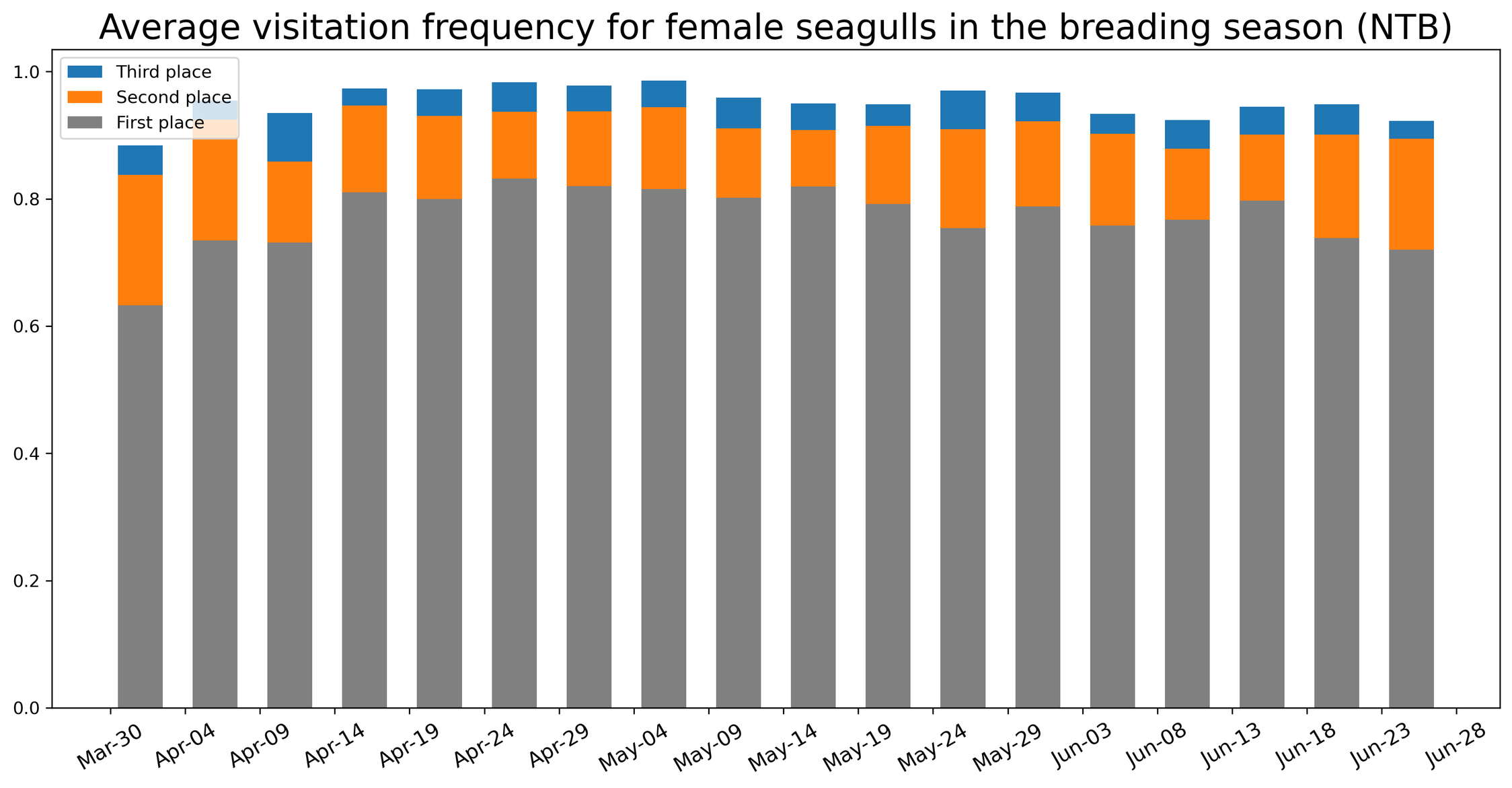

Supplement: S3 Fig — (TIF) [file pone.0286239.s003.tif]

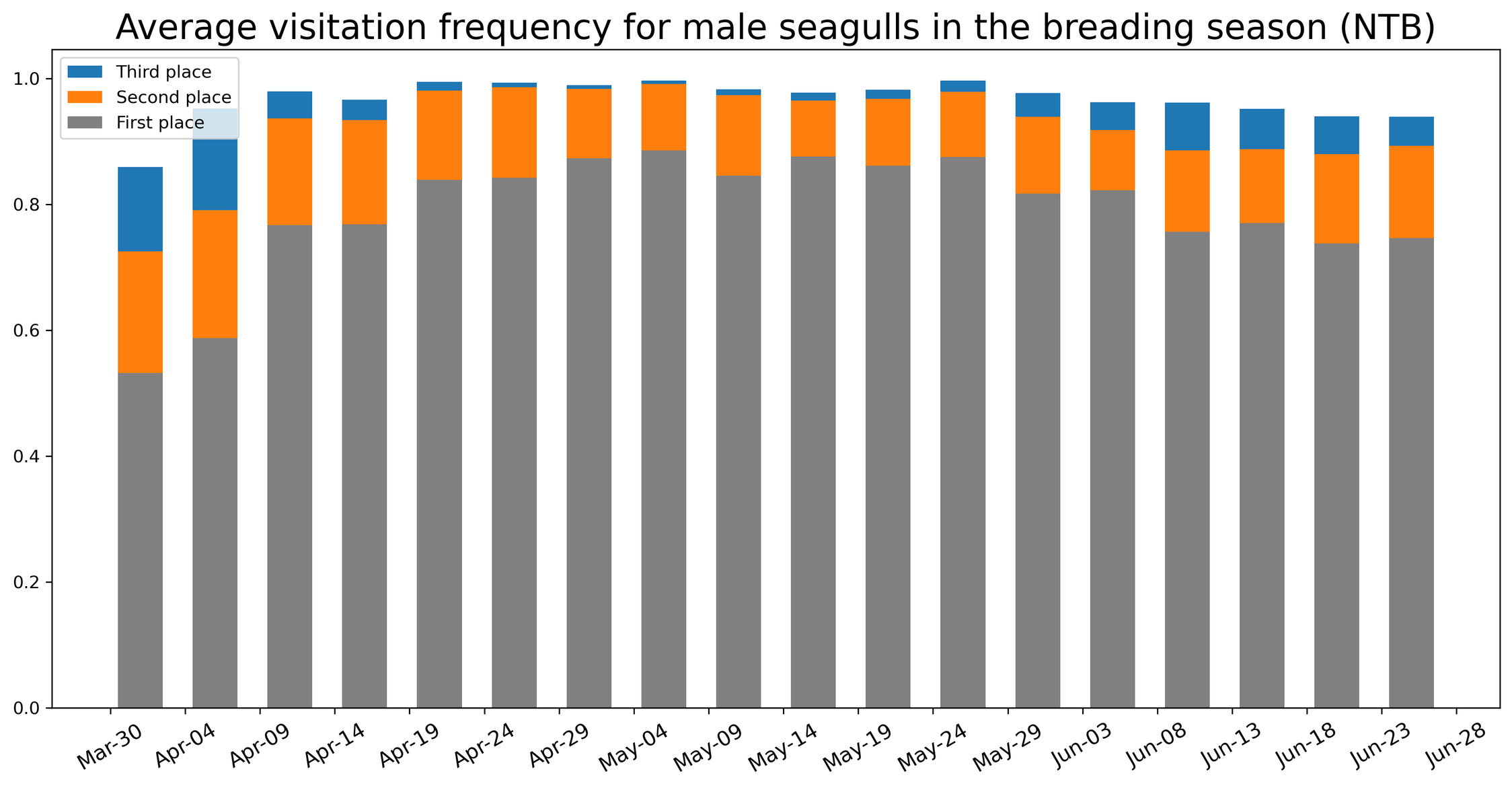

Supplement: S4 Fig — (TIF) [file pone.0286239.s004.tif]
